# Anti-Amyloid Monoclonal Antibodies in Early Alzheimer Disease: Lecanemab and Donanemab

**DOI:** 10.3390/neurolint18090171

**Published:** 2026-09-11

**Authors:** Ülkü Figen Demir, Fatmanur Karakuş Dilbaz

**Affiliations:** Department of Neurology, Faculty of Medicine, Istanbul Yeni Yüzyıl University, Istanbul 34010, Türkiye; fatmanur.karakusdilbaz@yeniyuzyil.edu.tr

**Keywords:** Alzheimer disease, amyloid beta-peptides, monoclonal antibodies, lecanemab, donanemab, anti-amyloid therapy, amyloid-related imaging abnormalities, APOE ε4, biomarkers, real-world evidence

## Abstract

Background/Objectives: Alzheimer disease (AD) causes progressive cognitive and functional loss and substantial caregiver and healthcare burden. Anti-amyloid monoclonal antibodies represent a shift toward biology-directed treatment in biomarker-confirmed early symptomatic AD, but modest clinical effects must be balanced against amyloid-related imaging abnormalities (ARIA), intensive monitoring, and implementation burden. Heterogeneity in trial populations, endpoints, dosing, stopping rules, and follow-up complicates interpretation. This narrative review critically integrates efficacy, safety, durability, biomarker, and implementation evidence for lecanemab and donanemab while preserving study-family relationships and avoiding unsupported cross-trial superiority claims. Methods: For this revised narrative review, a targeted PubMed/MEDLINE search covering the period from database inception was initially conducted before manuscript submission and was subsequently updated through 21 August 2026, supplemented by reference-list and citation tracking. Search terms combined Alzheimer disease with lecanemab, donanemab, anti-amyloid monoclonal antibody, ARIA, APOE, amyloid PET, open-label extension, real-world, clinical meaningfulness, implementation, and access. Sixty-two sources were purposively selected for a comprehensive narrative synthesis; no meta-analysis or formal certainty grading was performed. Results: Pivotal trials demonstrated statistically significant but modest average slowing of decline: Clarity AD showed a 0.45-point between-group difference in CDR-SB worsening at 18 months, and TRAILBLAZER-ALZ 2 showed a 3.25-point iADRS difference in the low/medium-tau population at 76 weeks. ARIA-E occurred in 12.6% of lecanemab-treated and 24.0% of donanemab-treated participants in the pivotal trials, with higher risk in APOE ε4 carriers. Extensions and biomarker analyses suggest persistent biological effects but are less secure for causal inference, and real-world evidence is currently more mature for lecanemab. Conclusions: Both antibodies substantially reduce amyloid and modestly slow average clinical decline in selected patients, but neither restores lost function, greater amyloid clearance does not establish greater individual benefit, and cross-trial superiority cannot be inferred. Treatment requires biomarker-guided selection, APOE-informed risk counseling, serial MRI, infusion and ARIA-management capacity, and shared decision-making that incorporates cost, access, and patient/caregiver burden.

## 1. Introduction

Alzheimer disease (AD) is the most common cause of dementia and a major source of progressive loss of independence, caregiver burden, and healthcare use worldwide [1,2]. For decades, pharmacologic management was predominantly symptomatic. The recent development of amyloid-β-directed monoclonal antibodies has shifted the therapeutic framework toward disease modification in biomarker-confirmed early symptomatic AD, while also introducing new questions about clinical meaningfulness, safety, resource use, and equitable access [3,4,5,6,7].

AD is biologically characterized by amyloid-β (Aβ) and tau pathology. Aβ is generated by proteolytic processing of amyloid precursor protein and can aggregate from soluble oligomeric and protofibrillar species into fibrils and extracellular plaques, with amyloid accumulation generally preceding overt clinical symptoms. Although amyloid burden alone does not explain the full clinical phenotype, its early position in the disease process and the clinical effects observed with recent plaque-lowering therapies provide a rationale for targeting Aβ as a disease-modifying strategy [3].

Lecanemab and donanemab are anti-amyloid monoclonal antibodies developed for patients with biomarker-confirmed early symptomatic Alzheimer disease, encompassing mild cognitive impairment due to AD and mild AD dementia. Lecanemab preferentially binds soluble aggregated amyloid-β species, including protofibrils, whereas donanemab targets an N-terminally modified pyroglutamate amyloid epitope enriched in established plaques [3,8,9,10]. Their pivotal programs established robust plaque removal and statistically significant slowing of cognitive-functional decline, but not symptomatic improvement or reversal of disease [11,12,13].

Interpretation is complicated by different eligibility criteria, dosing schedules, tau-enrichment strategies, outcome scales, amyloid-based stopping rules, and follow-up periods. Cross-trial percentage-slowing estimates are especially sensitive to the rate of decline in each placebo group and therefore do not establish comparative superiority. Network meta-analyses and indirect comparisons can generate hypotheses, but their rankings depend on assumptions of exchangeability that are difficult to satisfy across these programs [4,5,14].

Safety is central to the benefit–risk balance. Amyloid-related imaging abnormalities (ARIA) include vasogenic edema or sulcal effusion (ARIA-E) and microhemorrhage, superficial siderosis, or other hemosiderin deposition (ARIA-H). Most detected events are asymptomatic, but symptomatic and serious events occur, and risk varies with APOE ε4 genotype and baseline cerebrovascular imaging features [15,16,17]. Treatment also entails frequent intravenous infusions, serial magnetic resonance imaging (MRI), management of infusion reactions, and substantial diagnostic and operational requirements [6,7,18,19].

The unresolved clinical problem is not simply whether anti-amyloid antibodies remove plaque, but how modest group-average slowing of decline should be interpreted across heterogeneous trials and translated into individual benefit–risk decisions. Randomized trials, open-label extensions, post hoc analyses, pharmacometric models, and real-world cohorts answer different questions and provide different levels of causal certainty. Accordingly, this comprehensive narrative review integrates evidence by study family, separates pivotal randomized findings from complementary evidence, and aims to (1) summarize principal efficacy and safety findings; (2) clarify the clinical meaning and limitations of reported effect sizes; (3) distinguish target engagement from individual clinical benefit; and (4) identify implementation, access, and evidence gaps relevant to contemporary practice.

## 2. Methods

### 2.1. Review Design and Source Selection

This article was designed as a comprehensive narrative review rather than a systematic, scoping, or meta-analytic review. For this revised narrative review, a targeted PubMed/MEDLINE search covering the period from database inception was initially conducted before manuscript submission and was subsequently updated through 21 August 2026, supplemented by reference-list and citation tracking. Search strings combined “Alzheimer disease” with “lecanemab,” “donanemab,” “anti-amyloid monoclonal antibody,” “amyloid-related imaging abnormalities” or “ARIA,” “APOE,” “amyloid PET,” “open-label extension,” “real-world,” “clinical meaningfulness,” “implementation,” “MRI,” and “cost” or “access.” Reference lists of pivotal trials, major reviews, appropriate-use recommendations, and regulatory documents were additionally hand-searched to identify clinically consequential sources.

Eligible sources were English-language full-text reports addressing efficacy, safety, durability, biological effects, patient selection, or clinical implementation of lecanemab or donanemab in early symptomatic AD. Priority was given to pivotal randomized trials, full extension reports, prespecified or clinically informative secondary analyses, pooled safety analyses, pharmacometric studies, real-world cohorts, and selected contextual or regulatory sources. Conference abstracts without a full report, non-human studies, duplicate reports, studies of other anti-amyloid agents without direct contextual relevance, and secondary publications that did not add distinct clinical, safety, biomarker, or implementation information were not prioritized. The final evidence set comprised 62 sources. Selection was purposive rather than exhaustive, consistent with the narrative-review design. The selected literature was considered adequate for the intended narrative scope because it covered each prespecified evidence domain—pivotal randomized efficacy and safety, extensions and biomarker analyses, clinical meaningfulness, implementation, regulatory guidance, and contemporary real-world experience—while avoiding redundant secondary reports from overlapping trial populations.

No protocol was registered, screening was not performed in duplicate, and no PRISMA flow diagram, formal study-level risk-of-bias tool, or certainty-of-evidence grading was used. These choices reflect the interpretive purpose of the review and are acknowledged as limitations rather than features of a systematic review.

### 2.2. Evidence Classification and Study-Family Linkage

Evidence was classified as (1) randomized or prospective clinical evidence; (2) open-label extension, subgroup, biomarker, pharmacometric, or pooled safety evidence; (3) real-world cohort or implementation evidence; or (4) contextual evidence, including reviews, commentaries, policy analyses, protocols, and case reports. Secondary publications were linked to Study 201, Clarity AD, TRAILBLAZER-ALZ, or TRAILBLAZER-ALZ 2, as applicable, so that overlapping participants and events were not interpreted as independent cohorts.

For each publication, the review considered study design, population, sample size, intervention, comparator, duration, outcome measures, principal efficacy and safety findings, methodological limitations, and funding or conflicts of interest when reported. Full trial reports were used as the primary source for numerical estimates, with secondary analyses used to address durability, biomarkers, risk factors, and implementation.

### 2.3. Narrative Synthesis

Randomized, placebo-controlled results were prioritized for causal interpretation of efficacy and core safety. Extensions, secondary analyses, pharmacometric studies, and observational cohorts were used to assess durability, mechanisms, risk factors, feasibility, and applicability. Exact denominators, time points, and uncertainty intervals were reported when available, and cross-trial percentage estimates were not treated as head-to-head comparisons.

Because the included studies differed in eligibility criteria, treatment schedules, outcome scales, follow-up duration, and analytical design, findings were synthesized descriptively rather than pooled. No de novo meta-analysis, formal study-level risk-of-bias assessment, or certainty-of-evidence grading was performed.

## 3. Results

### 3.1. Evidence Architecture

The reviewed literature contains a small number of parent trials and a much larger number of overlapping secondary reports. The principal efficacy evidence derives from lecanemab Study 201 and Clarity AD and from donanemab TRAILBLAZER-ALZ and TRAILBLAZER-ALZ 2 [8,11,12,13]. Other reports extend or reanalyze these populations rather than contribute new randomized participants. Real-world lecanemab cohorts add information about implementation and short-term safety but generally lack randomized concurrent controls and have incomplete follow-up. Published real-world outcome evidence remains substantially more extensive for lecanemab than for donanemab; this asymmetry reflects different approval and adoption timelines and should not be interpreted as evidence of comparative superiority. The evidence architecture and its role in the synthesis are summarized in Table 1.

### 3.2. Lecanemab Randomized Evidence

Study 201 was an 18-month phase 2b, double-blind, Bayesian dose-finding trial. Of 854 treated participants, 609 received lecanemab and 245 received placebo [8]. The prespecified 12-month primary Bayesian decision criterion was not met: the 10 mg/kg biweekly regimen had a 64% probability of achieving at least 25% slowing on the Alzheimer Disease Composite Score, below the required 80% threshold [8,20]. At 18 months, prespecified secondary analyses favored 10 mg/kg biweekly, including estimated slowing of 27% by the Bayesian analysis and 30% by the frequentist analysis on the Alzheimer Disease Composite Score. Interpretation is constrained by multiplicity and a protocol change that excluded APOE ε4 carriers from the highest-dose arms, producing genotype imbalance [8,20].

Clarity AD randomized 1795 participants with early symptomatic AD to lecanemab 10 mg/kg every 2 weeks (*n* = 898) or placebo (*n* = 897) for 18 months [11]. Mean CDR-SB worsening was 1.21 with lecanemab and 1.66 with placebo, an adjusted difference of −0.45 points (95% CI, −0.67 to −0.23; *p* < 0.001). Prespecified secondary outcomes also favored lecanemab: the adjusted between-group differences were −1.44 points on ADAS-Cog14 (95% CI, −2.27 to −0.61), −0.050 on the Alzheimer Disease Composite Score (95% CI, −0.074 to −0.027), and +2.0 points on the ADCS-MCI-ADL (95% CI, 1.2–2.8) [11]. These are group-average differences in decline, not improvements from baseline.

In the Clarity AD amyloid-PET substudy, the adjusted mean change at 18 months was −55.48 centiloids with lecanemab and +3.64 with placebo, yielding a between-group difference of −59.12 centiloids (95% CI, −62.64 to −55.60) [11]. Biomarker analyses support downstream changes in soluble amyloid and tau pathways, but mediation of clinical benefit by any single biomarker remains inferential [11,21]. Principal efficacy findings from the pivotal randomized trials are summarized in Table 2.

### 3.3. Donanemab Randomized Evidence

Early dose-escalation and phase 1b studies established that monthly donanemab could produce substantial plaque reduction and informed later dosing, but they were not designed to establish clinical efficacy [9,10]. In the phase 2 TRAILBLAZER-ALZ trial, the principal two-arm efficacy population comprised 257 participants (131 donanemab and 126 placebo; 125 placebo participants contributed to the modified intention-to-treat analysis) [12]. At 76 weeks, mean change in the integrated Alzheimer Disease Rating Scale (iADRS) was −6.86 with donanemab and −10.06 with placebo, a difference of 3.20 points (95% CI, 0.12–6.27; *p* = 0.04). Most secondary clinical outcomes did not show a substantial between-group difference, and the small, tau-enriched sample limits precision [12].

TRAILBLAZER-ALZ 2 randomized 1736 participants at 277 sites in eight countries to donanemab (*n* = 860) or placebo (*n* = 876) for 76 weeks [13]. In the low/medium-tau population, iADRS change was −6.02 versus −9.27, a difference of 3.25 points (95% CI, 1.88–4.62), corresponding to 35.1% less decline relative to placebo. In the combined low/medium- plus high-tau population, iADRS change was −10.19 versus −13.11, a difference of 2.92 points (95% CI, 1.51–4.33), corresponding to 22.3% less decline [13].

CDR-SB differences at week 76 were −0.67 points (95% CI, −0.95 to −0.40) in the low/medium-tau population and −0.70 points (95% CI, −0.95 to −0.45) in the combined population [13]. Amyloid clearance below 24.1 centiloids was observed in 80% of low/medium-tau and 76% of combined donanemab-treated participants by week 76 [13]. The protocol allowed blinded conversion to placebo after prespecified amyloid thresholds were reached, so treatment exposure varied.

Post hoc analyses consistently suggest larger relative effects in participants with lower baseline tau or earlier modeled disease stage, but these analyses should be interpreted as effect-modification hypotheses rather than proof that a specific biomarker threshold identifies an individual responder [22]. A Japanese subgroup (*n* = 88) showed directionally consistent effects but wide uncertainty [23]; the Clarity AD Asian regional analysis was likewise descriptive and not powered for a definitive regional interaction [24]. European-eligible analyses applied population restrictions and missing-data methods not used in the primary report [25].

### 3.4. Safety and Tolerability

In Clarity AD, infusion reactions occurred in 26.4% of lecanemab-treated participants and 7.4% of placebo recipients. ARIA-E occurred in 12.6% versus 1.7%, symptomatic ARIA-E in 2.8% of lecanemab-treated participants, and ARIA-H in 17.3% versus 9.0%; symptomatic ARIA-H occurred in 0.7% versus 0.2% [11]. Serious adverse events occurred in 14.0% versus 11.3%, and adverse events led to discontinuation of trial drug in 6.9% versus 2.9% [11]. Deaths occurred in 0.7% and 0.8%, respectively, with none judged treatment-related by investigators during the blinded core [11].

ARIA-E risk with lecanemab varied markedly by APOE ε4 genotype in Clarity AD: 5.4% in noncarriers, 10.9% in heterozygotes, and 32.6% in homozygotes [11]. The combined core-plus-extension safety analysis included 1,612 lecanemab-exposed participants and reported four deaths considered possibly related to treatment during the open-label extension. Two lecanemab-treated participants had concurrent intracerebral hemorrhage, one after tissue plasminogen activator and one while receiving anticoagulant therapy [16]. This uncontrolled extension enlarges safety exposure but cannot provide a randomized comparison.

In TRAILBLAZER-ALZ 2, MRI-detected ARIA-E occurred in 24.0% of donanemab-treated participants and 2.1% of placebo recipients; symptomatic ARIA-E occurred in 6.1% and 0.1%, respectively [13]. ARIA-H occurred in 31.4% versus 13.6%. Infusion reactions occurred in 8.7% versus 0.5%, serious adverse events in 17.4% versus 15.8%, and adverse events led to discontinuation of treatment in 13.1% versus 4.3% [13]. Overall deaths in the safety population were 16/853 (1.9%) with donanemab and 10/874 (1.1%) with placebo; among these, 3 (0.4%) and 1 (0.1%), respectively, were considered treatment-related in the primary TRAILBLAZER-ALZ 2 report [13].

Donanemab-associated ARIA-E also showed an APOE ε4 gradient in TRAILBLAZER-ALZ 2: 15.7% in noncarriers, 22.8% in heterozygotes, and 40.6% in homozygotes [13]. A pooled analysis of 3,030 participants across randomized and open-label donanemab datasets identified APOE ε4 allele count, baseline microhemorrhage burden, cortical superficial siderosis, higher baseline amyloid burden, and higher mean arterial pressure as independent risk markers for ARIA-E [17]. A separate pooled analysis characterized donanemab infusion reactions as predominantly early and manageable, while emphasizing the need for observation and treatment protocols [26].

TRAILBLAZER-ALZ 6 tested a more gradual donanemab titration. At 76 weeks, ARIA-E occurred in 15.6% with modified titration and 24.2% with standard dosing; symptomatic ARIA-E occurred in 2.8% and 4.8%, respectively, while amyloid lowering was maintained [27]. These results support titration as a risk-mitigation strategy for donanemab, but they do not eliminate the need for genotype-informed counseling and MRI monitoring.

Clinically, ARIA-E represents vasogenic edema or sulcal effusion, whereas ARIA-H includes treatment-emergent microhemorrhage and/or superficial siderosis. Many events are asymptomatic and resolve radiographically, but symptomatic ARIA may present with headache, confusion, visual symptoms, dizziness, gait disturbance, seizures, or focal neurologic deficits and can rarely be serious or fatal. Typical management includes prompt clinical assessment and MRI for suggestive symptoms, temporary treatment interruption for symptomatic or radiographically moderate-to-severe ARIA, repeat MRI until stabilization or resolution, and individualized decisions about resumption; severe presentations require urgent neurologic management and some protocols use corticosteroids for severe ARIA-E [13,16,17,28,29]. Infusion reactions are usually early and mild-to-moderate; management may include slowing or interrupting the infusion, symptomatic therapy, and premedication for subsequent doses when appropriate [26,28,29]. Selected safety outcomes from the pivotal randomized trials are summarized in Table 3.

### 3.5. Extension, Biomarker, and Pharmacometric Evidence

The Clarity AD open-label extension reported maintained separation between early-start and delayed-start cohorts through 36 months [30]. Because all participants received lecanemab after the blinded phase, long-term comparisons rely on delayed-start contrasts and modeled or external natural-history assumptions. These analyses are compatible with durable benefit but do not preserve the original randomized placebo comparison. Donanemab long-term-extension data likewise suggest persistence of clinical separation after treatment-course completion, but delayed-start and external-control comparisons do not retain the same causal strength as the blinded placebo-controlled phase [31].

Donanemab biomarker analyses found early reductions in plasma p-tau217 and glial fibrillary acidic protein and associations between biomarker change and amyloid reduction [32]. A TRAILBLAZER-ALZ 2 secondary analysis associated lower posttreatment amyloid levels with slower subsequent clinical decline, but residual confounding and postrandomization selection preclude interpreting the association as a validated individual treatment target [33]. More generally, the magnitude of amyloid reduction is a pharmacodynamic measure of target engagement; greater plaque clearance should not be interpreted as proof of greater clinical benefit for an individual patient.

Population pharmacokinetic and exposure-response analyses characterized donanemab plaque reduction and safety and lecanemab exposure-related ARIA risk and pharmacokinetics [34,35]. Semimechanistic modeling additionally linked amyloid PET burden and treatment-related amyloid reduction with modeled CDR-SB trajectories [36]. These models synthesize repeated measurements from parent trials; they do not constitute independent demonstrations of clinical efficacy. Trial-level analyses have reported associations between greater amyloid reduction, greater average clinical response, and higher ARIA-E rates; however, such aggregate associations do not establish individual-level clinical benefit or mediation [37]. Similarly, exploratory analyses of brain volume loss after plaque-removing therapy identify an imaging signal whose mechanism and clinical meaning remain unsettled [38,39].

### 3.6. Real-World Evidence and Evidence-Maturity Gap

Real-world cohorts demonstrate that lecanemab can be implemented in academic, specialty, private-practice, and multicenter settings, but they also show substantial variation in selection, follow-up, cognitive instruments, MRI schedules, and adverse-event ascertainment [40,41,42,43,44,45,46,47,48,49]. Most follow-up was short, and cognitive change without a randomized concurrent control cannot distinguish a treatment effect from natural history, practice effects, regression to the mean, or selection and attrition. At the search cutoff, lecanemab had the larger body of published practice-based safety and feasibility data, whereas donanemab real-world outcome literature was only beginning to emerge.

In a Washington University specialty memory clinic, 234 patients initiated lecanemab; among 194 at risk for ARIA during the observation period, 42 (22%) developed ARIA, including 29 (15%) with ARIA-E with or without ARIA-H and 13 (6.7%) with isolated ARIA-H. Eleven patients (5.7%) had symptomatic ARIA, including two with clinically severe symptoms, and no macrohemorrhage or death occurred [43]. A two-clinic US report of 165 patients found ARIA-E in 8% and ARIA-H in 7%, with three symptomatic cases requiring immediate medical attention; the report lacked 12-month efficacy data [44].

A Tel Aviv cohort screened 169 patients and initiated lecanemab in 86. ARIA occurred in 18.6%, infusion reactions in 22.1%, and 19.8% discontinued for ARIA, financial barriers, comorbidity, or preference [42]. MMSE declined over six months in the intention-to-treat population, whereas change among the 31 patients reaching 12 months was not statistically significant; these findings should not be interpreted as a controlled efficacy estimate [42].

Chinese cohorts reported 6- to 7-month amyloid and plasma-biomarker changes, with ARIA estimates ranging from 7.1% in a 42-patient cohort to 17.65% in a 68-patient cohort and 9.2% in a 261-patient multicenter cohort [40,41,45]. The 261-patient study used a matched Alzheimer’s Disease Neuroimaging Initiative cohort and reported a mean amyloid change of −22.1 centiloids, 29.2% conversion to amyloid negativity, infusion reactions in 11.1%, and symptomatic ARIA in 1.6% [45]. The external comparator, short duration, optional imaging, and inclusion of participants beyond mild dementia limit causal and label-concordant interpretation.

Other Asian practice cohorts reinforce heterogeneity. In an Eastern China cohort of 76 treated patients, 11 developed ARIA, all asymptomatic, and 14 (18.4%) had infusion-related effects [46]. In a Japanese clinical-practice cohort, 12 of 71 patients with MRI before the 14th infusion had ARIA (16.9%); higher baseline CSF p-tau181 was associated with ARIA and subsequent cognitive decline, but the proposed cutoff requires external validation [47]. A separate 120-patient Japanese cohort reported ARIA in 20%, comprising ARIA-E with or without ARIA-H in 4% and isolated ARIA-H in 16% [48]. In a 139-patient Northwestern China registry, all recorded infusion reactions were grade 1; hypertension and higher Fazekas score were associated with reactions, but event frequency and predictors require replication [49].

Early donanemab real-world evidence is much less mature. A 2026 Italian tertiary-memory-center cohort reported 29 treatment courses (20 donanemab and 9 lecanemab) with short follow-up under structured MRI monitoring; the findings were descriptive and explicitly not intended for between-drug comparison [50]. Thus, the imbalance in this review—multiple lecanemab cohorts versus sparse donanemab practice data—reflects the relative maturity and publication timing of the evidence base rather than preferential weighting of one treatment. Selected real-world anti-amyloid cohorts are summarized in Table 4.

### 3.7. Clinical Interpretation and Indirect Comparison

The pivotal trials support the conclusion that both drugs slow average decline relative to placebo in selected patients with early symptomatic AD. They do not support a direct lecanemab-versus-donanemab efficacy or safety comparison. The drugs were evaluated in different populations, over different durations, with different primary scales, tau requirements, dosing and stopping rules, and adverse-event monitoring. Comparing 27% and 35% relative slowing, or any other cross-trial percentages, as if they were head-to-head effects is methodologically invalid [5,11,13,14].

The absolute between-group differences were small relative to the full range of the outcome scales and require clinical context. CDR-SB ranges from 0 to 18, with higher scores indicating greater impairment, whereas iADRS ranges from 0 to 144, with lower scores indicating greater impairment [11,13]. In practical terms, the 0.45-point lower CDR-SB worsening with lecanemab at 18 months and the 3.25-point smaller iADRS decline with donanemab in the low/medium-tau population at 76 weeks mean that treated groups accumulated less average cognitive-functional worsening than placebo; they do not mean that patients improved from baseline or that every individual experienced the average difference. Published estimates of meaningful within-patient change vary by disease stage and method, and applying such thresholds directly to between-group trial effects remains debated [4,5,14,51,52,53,54]. Accordingly, these findings are best communicated to patients and caregivers as modest average slowing of progression with uncertain individual perceptibility, supplemented where available by time-to-progression, responder, and patient/caregiver-centered analyses rather than by a single numerical threshold.

## 4. Discussion

Across the evidence reviewed, the most defensible conclusion is narrow but clinically important: lecanemab and donanemab produce large reductions in cerebral amyloid and modest average slowing of cognitive-functional decline over approximately 18 months in trial-eligible patients with biomarker-confirmed early symptomatic AD [11,13,55]. The trials do not show restoration of cognition or independence, and their results should not be extrapolated to moderate or severe dementia, unconfirmed amyloid pathology, or patients with excluded hemorrhagic MRI findings. Large biomarker effects should therefore be interpreted as evidence of target engagement, not as a surrogate proof that a patient with greater plaque removal will necessarily obtain greater cognitive or functional benefit.

The safety burden is material. ARIA risk is concentrated early in treatment and rises with APOE ε4 allele count and baseline hemorrhagic imaging markers [11,13,17]. Although most ARIA is radiographic and mild or moderate, symptomatic events, serious events, intracerebral hemorrhage, and treatment-associated deaths have been reported [13,16,17]. The pertinent clinical question is therefore not whether ARIA is usually asymptomatic, but whether a specific patient’s expected delay in decline justifies the individualized neurologic risk and treatment burden.

APOE genotyping informs, but does not determine, the decision. Homozygotes had the highest ARIA-E incidence in both pivotal programs; noncarriers were not risk-free [11,13]. Baseline microhemorrhages, superficial siderosis, blood pressure, antithrombotic exposure, access to urgent MRI, and the consequences of thrombolysis or anticoagulation during treatment also require consideration [16,17,28,29]. Genotype subgroup estimates and emerging MRI or fluid-biomarker predictors should not yet be treated as validated individual-response algorithms [47,56].

Implementation studies show that treatment pathways require coordination among cognitive assessment, amyloid confirmation, APOE counseling, MRI eligibility review, infusion services, adverse-event triage, and longitudinal monitoring [19,28,29,43,44]. Practical burden extends beyond adverse events: lecanemab has traditionally required infusions every 2 weeks during the initial treatment phase, whereas donanemab is administered every 4 weeks; both require scheduled and symptom-triggered MRI, and donanemab may require amyloid PET to support treatment-course completion decisions. Infusion-chair time, travel, post-infusion observation, urgent imaging capacity, genetic counseling, treatment interruptions, cost and insurance coverage, and time contributed by patients and care partners can all affect persistence and real-world value [6,7,28,29,42,57,58,59,60]. These operational demands require specialized radiology, infusion, pharmacy, and emergency pathways and may narrow real-world eligibility or amplify inequities that are not captured by trial efficacy estimates.

Digital monitoring may complement this clinic-based pathway by providing more frequent and ecologically relevant measures of cognition and everyday function between scheduled visits. Smartphone- or tablet-based cognitive assessments, speech and motor measures, and wearable-derived activity or sleep data could help characterize longitudinal change and prompt focused clinical reassessment, although validation, standardization, adherence, privacy, and interpretability remain important limitations [61]. In parallel, structured real-world registries and multicenter cohorts can link treatment exposure with serial cognitive-functional measures, MRI/ARIA events, biomarkers, discontinuation, and healthcare utilization across broader populations, thereby helping assess effectiveness and safety beyond trial-eligible cohorts and supporting more individualized treatment monitoring [19,40,41,42,43,44,45,46,47,48,49,50]. These approaches should complement rather than replace standardized clinical assessment and established MRI safety surveillance.

Longer-term evidence remains promising but less secure than the blinded core trials. Open-label extensions suggest continued separation from delayed-start or modeled comparators, while posttreatment and biomarker analyses support biological plausibility [30,31,32,33]. Nevertheless, attrition, selective continuation, treatment switching, and reliance on external controls weaken causal inference. Long-term randomized or otherwise robust comparative follow-up, patient-centered outcomes, and diverse real-world registries remain priorities. The evidence base is also temporally asymmetric: lecanemab currently has broader published real-world follow-up, whereas donanemab implementation data remain comparatively early and sparse. Evidence-informed clinical interpretations for practice are summarized in Table 5.

A strength of this review is its explicit linkage of secondary publications to parent trial families, separation of randomized evidence from extensions and modeled or observational analyses, and integration of efficacy, safety, biomarker, pharmacometric, implementation, and real-world perspectives. This structure helps avoid double-counting overlapping populations and, importantly, avoids converting cross-trial percentage differences or amyloid-reduction magnitudes into unsupported claims of comparative superiority.

Despite the targeted search strategy, this narrative review remains susceptible to selection bias and does not establish that all relevant publications were identified. No protocol was registered, screening was not performed in duplicate, and formal study-level risk-of-bias or certainty-of-evidence assessments were not conducted. In addition, several publications were secondary, post hoc, modeled, or overlapping reports from the same parent trials.

The pivotal trials and many secondary analyses were sponsor-funded, and numerous authors were sponsor employees or shareholders. This does not invalidate the findings, but it strengthens the need to distinguish prespecified randomized analyses from post hoc, modeled, and sponsor-interpreted extensions. Real-world studies were mostly small, short, and observational, often with incomplete outcome data.

Finally, the review did not pool results or perform indirect treatment comparisons. That choice limits numerical summary but avoids combining nonexchangeable populations, outcomes, and follow-up periods. Regulatory indications and monitoring requirements are not globally uniform and continue to evolve; trial protocols should therefore not be presented as universal clinical rules.

For example, current U.S. prescribing information permits treatment of biomarker-confirmed mild cognitive impairment or mild dementia due to AD while recommending APOE ε4 testing to inform ARIA risk; European indications for both lecanemab and donanemab restrict use to APOE ε4 noncarriers or heterozygotes [57,58,59,60]. MRI schedules also differ: the current U.S. lecanemab label specifies baseline MRI and scans before the 3rd, 5th, 7th, and 14th infusions, whereas the donanemab label specifies baseline MRI and scans before the 2nd, 3rd, 4th, and 7th infusions [57,58]. Donanemab labeling allows consideration of stopping after amyloid PET demonstrates minimal plaque levels, while U.S. lecanemab labeling allows continued or maintenance therapy after 18 months [57,58]. Eligibility, APOE testing, MRI timing, treatment duration, and stopping rules in this review should therefore be interpreted against current local product information rather than generalized across jurisdictions.

For practice, appropriate use requires confirmation of amyloid pathology, clinical staging within the studied early symptomatic range, baseline MRI assessment, APOE-informed counseling, review of cerebrovascular and antithrombotic risks, and capacity for scheduled and symptom-triggered MRI. Clinical applicability is limited by the selected nature of pivotal trial populations, underrepresentation of several racial and ethnic groups, comorbidity exclusions, and the infrastructure required to deliver treatment safely. Shared decision-making should present absolute scale differences and uncertainty alongside relative slowing, ARIA and infusion-reaction risks, treatment frequency and duration, MRI and PET requirements, cost and access, and the practical workload carried by patients and caregivers [28,29,57,58,59,60].

Research priorities include longer controlled follow-up; head-to-head or otherwise robust comparative-effectiveness studies; enrollment of more racially, ethnically, medically, and socioeconomically diverse populations; validated predictors of individual benefit and harm; patient- and caregiver-valued outcomes; and transparent evaluation of treatment duration, stopping rules, brain-volume changes, post-clearance trajectories, digital monitoring, and real-world registries [30,31,33,39,50,56,61]. Emerging MRI biomarkers such as DTI-ALPS and choroid plexus measures remain exploratory and require larger controlled validation [62].

## 5. Conclusions

Anti-amyloid monoclonal antibodies have shifted treatment of early Alzheimer disease from exclusively symptomatic management toward biology-directed disease modification. Across pivotal trials, lecanemab and donanemab produced substantial amyloid lowering and statistically significant, but modest, average slowing of cognitive-functional decline. These group-level effects should not be interpreted as restoration of lost function, proof that greater amyloid clearance yields greater individual benefit, or evidence that one antibody is superior to the other. Clinical use therefore depends on more than efficacy estimates. Amyloid confirmation, stage-appropriate patient selection, APOE-informed ARIA counseling, baseline and serial MRI, management of infusion reactions and ARIA, infusion capacity, cost and access, and patient/caregiver burden all shape the net value of treatment. Regulatory eligibility, MRI schedules, treatment duration, and stopping rules differ across jurisdictions and continue to evolve. Longer-term extensions and emerging real-world cohorts add important information but remain less secure for causal inference than blinded randomized trials; real-world evidence is also currently more mature for lecanemab than for donanemab. Future priorities include head-to-head or robust comparative-effectiveness studies, longer controlled follow-up, validated predictors of individual benefit and harm, diverse registries, patient- and caregiver-centered outcomes, and evidence-based stopping strategies. Until then, treatment decisions should be individualized through transparent shared decision-making that presents modest expected benefit alongside neurologic risk and practical burden.

## Figures and Tables

**Table 1:** Evidence architecture and role in the synthesis.

Evidence Layer	Principal Reports	What it Supports	Key Caution
Lecanemab randomized cores	Study 201; Clarity AD	Causal efficacy, amyloid lowering, 18-month safety	Study 201 primary 12-month decision criterion was not met; Clarity AD follow-up was 18 months
Donanemab randomized cores	Phase 1b/dose escalation; TRAILBLAZER-ALZ; TRAILBLAZER-ALZ 2; ALZ 6	Dose selection, causal efficacy, amyloid lowering, safety, titration	Different populations and purposes; early-phase studies do not establish clinical efficacy
Extensions and secondary analyses	Clarity AD OLE; regional, APOE, tau, biomarker, ARIA, and PK/PD reports	Durability, generalizability, mechanism, risk factors	Overlapping participants; many analyses are post hoc, open-label, modeled, or externally controlled
Real-world cohorts	Predominantly lecanemab practice cohorts; emerging mixed-agent experience	Feasibility, selection, ARIA, infusion reactions, discontinuation	Evidence maturity is greater for lecanemab; cohorts are mostly short, uncontrolled, and affected by incomplete follow-up
Contextual evidence	Systematic reviews, commentaries, policy analyses, protocols, case reports	Clinical meaningfulness, access, implementation, future research	Not counted as independent primary efficacy evidence

Abbreviations: OLE, open-label extension; PK/PD, pharmacokinetic/pharmacodynamic.

**Table 2:** Principal efficacy findings from pivotal randomized trials.

Trial	Population and Duration	Primary Clinical Result	Amyloid Result	Interpretive Limitation
Clarity AD (lecanemab)	*n* = 1795; early AD; 18 months; lecanemab 10 mg/kg every 2 weeks vs. placebo	CDR-SB change 1.21 vs. 1.66; difference −0.45 (95% CI, −0.67 to −0.23)	PET substudy: adjusted mean change −55.48 vs. +3.64 centiloids; difference −59.12	Selected trial population; modest absolute scale difference; 18-month horizon; plaque reduction does not establish individual clinical benefit
TRAILBLAZER-ALZ 2 (donanemab)	*n* = 1736; early symptomatic AD; 76 weeks; donanemab every 4 weeks vs. placebo	iADRS difference 3.25 (95% CI, 1.88–4.62) in low/medium tau; 2.92 (95% CI, 1.51–4.33) combined	Amyloid clearance <24.1 centiloids in 80% of low/medium tau and 76% combined	Tau enrichment and amyloid-based stopping; variable exposure; no head-to-head comparison; plaque clearance does not establish individual response

CDR-SB, Clinical Dementia Rating–Sum of Boxes; CI, confidence interval; iADRS, integrated Alzheimer Disease Rating Scale; PET, positron emission tomography. Because the trials differed in populations, endpoints, tau selection, treatment duration, dosing, and stopping rules, relative efficacy percentages and amyloid-lowering magnitudes are not head-to-head estimates and should not be used to infer comparative superiority.

**Table 3:** Selected safety outcomes in pivotal randomized trials.

Outcome	Clarity AD: Lecanemab vs. Placebo	TRAILBLAZER-ALZ 2: Donanemab vs. Placebo
ARIA-E	12.6% vs. 1.7%	24.0% vs. 2.1%
Symptomatic ARIA-E	2.8% vs. not separately reported in the principal table	6.1% vs. 0.1%
ARIA-H	17.3% vs. 9.0%	31.4% vs. 13.6%
Infusion reactions	26.4% vs. 7.4%	8.7% vs. 0.5%
Serious adverse events	14.0% vs. 11.3%	17.4% vs. 15.8%
Treatment discontinuation due to adverse event	6.9% vs. 2.9%	13.1% vs. 4.3%
Overall deaths/treatment-related deaths	Overall: 0.7% vs. 0.8%; treatment-related: 0% vs. 0%	Overall: 1.9% vs. 1.1%; treatment-related: 0.4% vs. 0.1%

Abbreviations: ARIA-E, amyloid-related imaging abnormalities with edema/effusion; ARIA-H, amyloid-related imaging abnormalities with hemorrhage/hemosiderin deposition. Definitions and ascertainment differed between programs; percentages are not head-to-head comparisons. For TRAILBLAZER-ALZ 2, overall mortality and investigator-judged treatment-related mortality are shown as separate outcomes using the primary JAMA safety population (donanemab *n* = 853; placebo *n* = 874) [13].

**Table 4:** Selected real-world anti-amyloid cohorts.

Study/Setting	Treated Sample and Follow-Up	Selected Findings	Principal Limitation
Paczynski et al. [43]; US specialty memory clinic	234 initiated; mean treatment 6.5 months	ARIA in 22% of 194 at risk; symptomatic ARIA 5.7%; infusion reactions 37%; no macrohemorrhage or death	Retrospective, single-center, short follow-up; safety denominator differed from initiated cohort
Bregman et al. [42]; Tel Aviv tertiary center	86 initiated; 53 reached 6 months; 31 reached 12 months	ARIA 18.6%; infusion reactions 22.1%; discontinuation 19.8%; heterogeneous MMSE trajectory	Small, uncontrolled, incomplete follow-up
Liao et al. [45]; 21 Chinese sites	261; 6 months; external matched ADNI comparator	Amyloid −22.1 centiloids; 29.2% amyloid-negative; ARIA 9.2% (1.6% symptomatic); infusion reactions 11.1%	External control, optional imaging, short follow-up, broader disease severity
Mao et al. [40]; PUMCH cohort	42 treated; 29 completed 6 months	Median amyloid reduction 30.9 centiloids; PET negativity 24.1%; asymptomatic mild ARIA 7.1%	Small cohort, attrition, matched observational controls
Shang et al. [46]; Eastern China	76 initiated; follow-up duration varied	11 ARIA events, all asymptomatic; infusion-related effects 18.4%; amyloid reduction at 12 months	Single center; few reached 12 months; no randomized comparator
Noguchi-Shinohara et al. [47]; Japanese practice	100 initiated; 71 had MRI before infusion 14	ARIA 16.9% in MRI-evaluable group; infusion reactions 6.0%; exploratory CSF p-tau181 risk marker	Selected MRI-evaluable denominator; proposed biomarker cutoff unvalidated
Shimasaki et al. [48]; Japanese center	120; up to 18 months; 81 completed 12 months	ARIA 20%: ARIA-E ± ARIA-H 4%, isolated ARIA-H 16%	Single-center observational study with incomplete longitudinal follow-up
Agosta et al. [50]; Italian tertiary memory center	29 treatment courses; lecanemab *n* = 9, donanemab *n* = 20; short-term follow-up	Structured MRI monitoring; early feasibility and safety data for both agents	Small, single-center, descriptive cohort; unbalanced groups; not designed for direct comparison

Abbreviations: ADNI, Alzheimer’s Disease Neuroimaging Initiative; ARIA, amyloid-related imaging abnormalities; CSF, cerebrospinal fluid; MMSE, Mini-Mental State Examination; PET, positron emission tomography.

**Table 5:** Evidence-informed interpretation for clinical use.

Question	Supported Conclusion	What Remains Uncertain
Do the drugs remove amyloid?	Yes; both produce large average reductions in trial populations.	The optimal individual posttreatment amyloid target, the relationship between plaque clearance and individual clinical benefit, and stopping strategy.
Do the drugs improve patients?	No restoration was shown; trials demonstrated less worsening than placebo.	Which individuals experience a noticeable or durable benefit.
Which drug is superior?	No conclusion can be drawn; no lecanemab–donanemab head-to-head clinical-outcome trial is available.	Comparative effectiveness and safety under common eligibility and monitoring.
Who is at greatest ARIA risk?	Risk rises with APOE ε4 allele count and baseline hemorrhagic imaging findings.	Validated multivariable prediction and management in medically complex populations.
Are long-term benefits established?	Extensions are compatible with continued benefit.	Magnitude under long-term randomized comparison and effects after stopping.
Does real-world evidence confirm efficacy?	It supports feasibility and characterizes short-term safety, with a more mature published evidence base for lecanemab than donanemab.	Causal effectiveness because most cohorts are uncontrolled, short, and not designed for head-to-head comparison.
Are implementation rules universal?	No. Eligibility, APOE requirements, MRI schedules, treatment duration, and stopping rules vary by jurisdiction and label.	How evolving national labels and reimbursement policies affect comparative uptake and outcomes.

## Data Availability

No new data were created or analyzed in this study. Data sharing is not applicable to this article.
